# Supplementary figures and images for: A comparative analysis of the rhizosphere microbial communities among three species of the Salix genus
Source: PeerJ. 2025 Mar 28;13:e19182. doi: 10.7717/peerj.19182 (PMC11956769; doi:10.7717/peerj.19182)

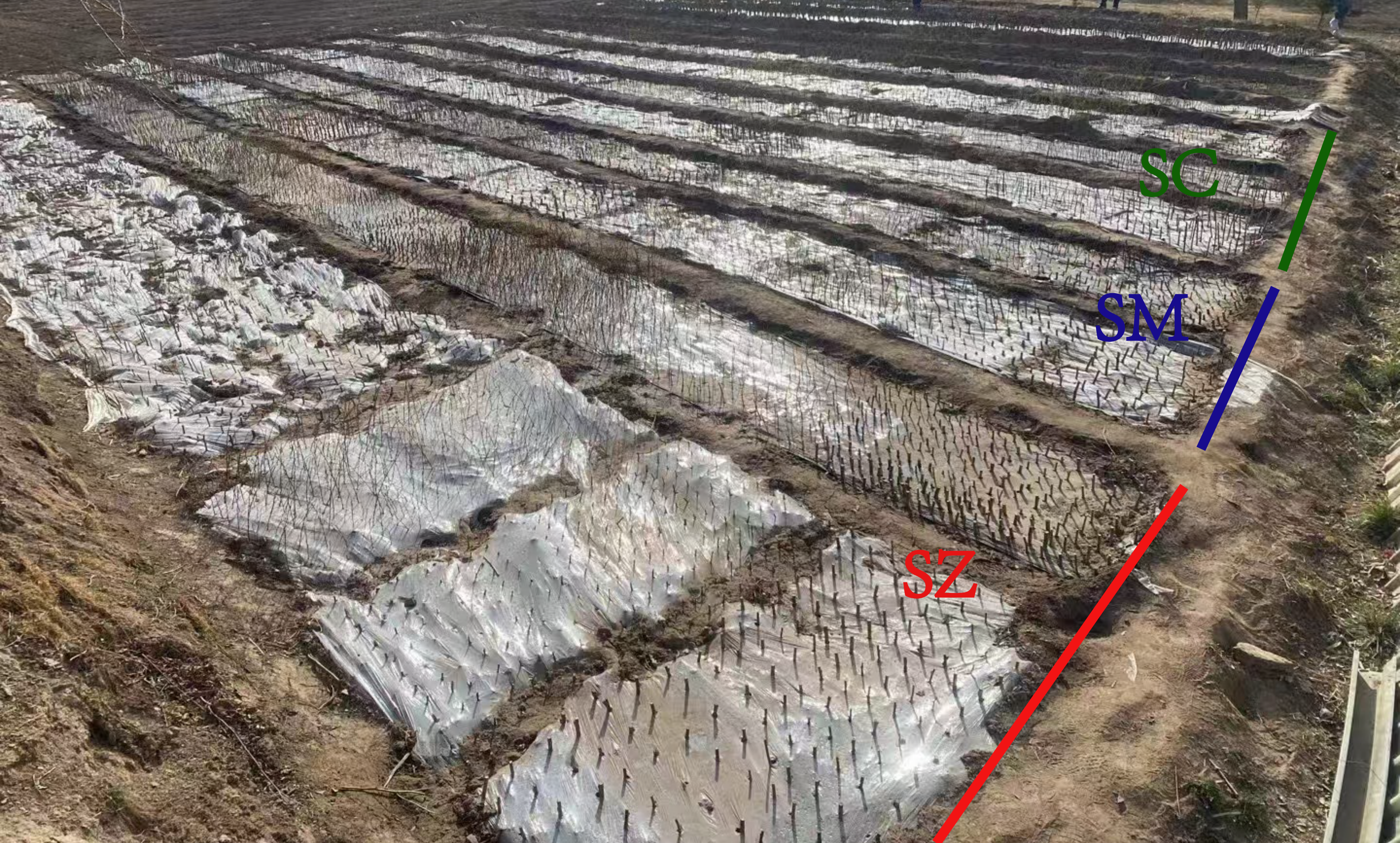

SC

SM

SZ

Supplement: Supplemental Information 1 [file peerj-13-19182-s001.pdf]

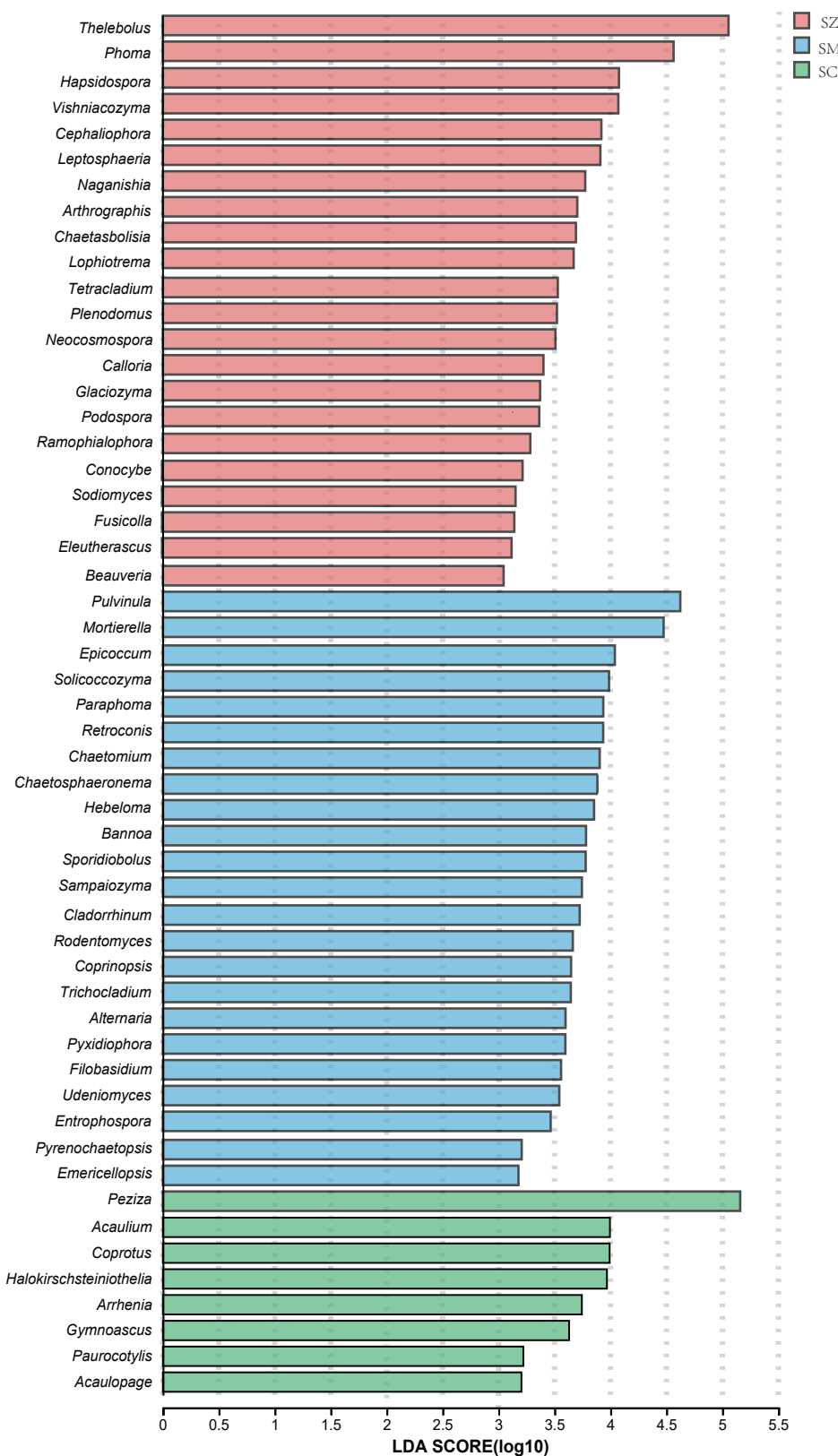

Supplement: Supplemental Information 2 — SZ, SM, and SC represent Salix zangica, Salix myrtilllacea, and Salix cheilophila samples, respectively. [file peerj-13-19182-s002.pdf]

**A**

Gram Negative

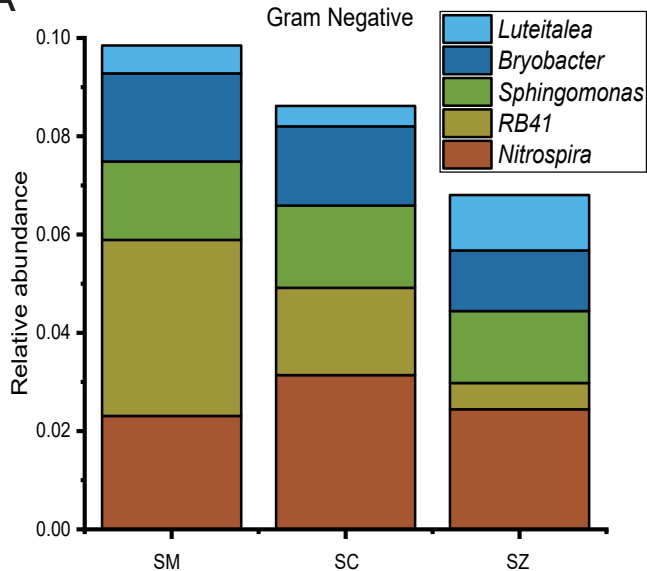**B**

Gram Positive

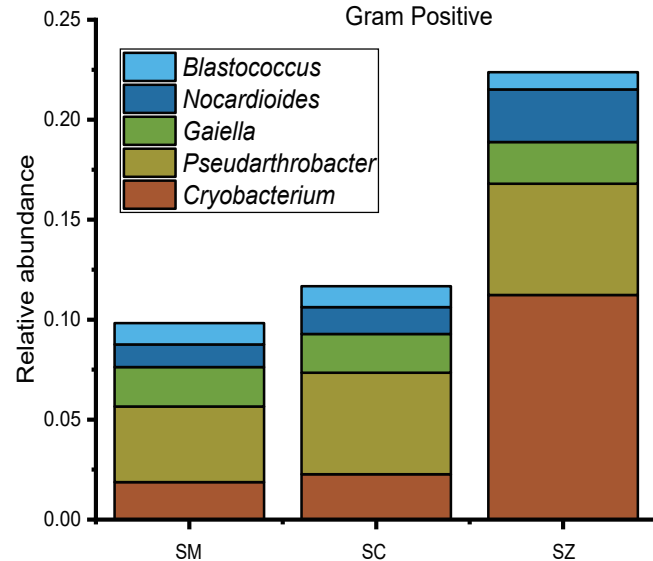**C**

Potentially Pathogenic

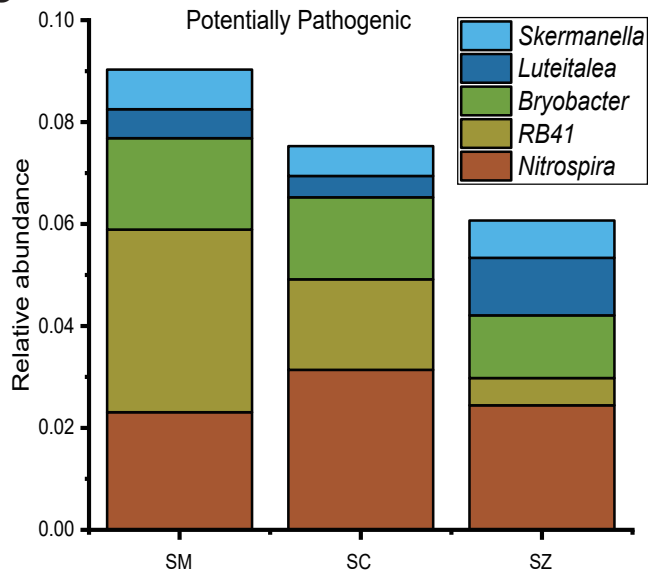**D**

Stress Tolerant

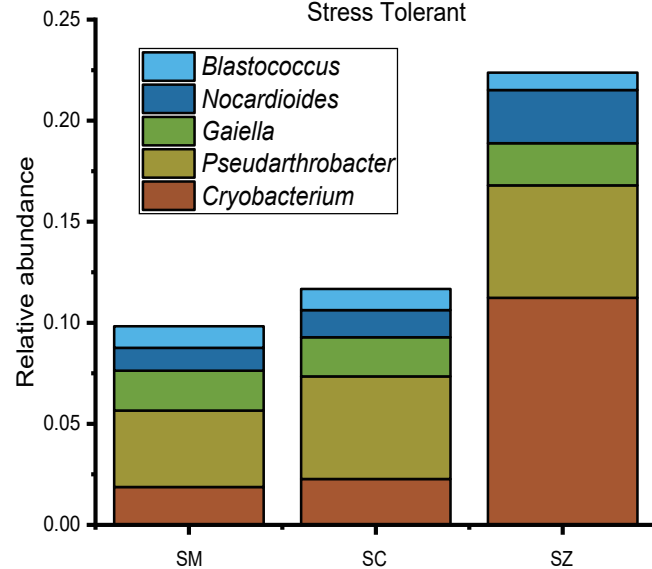

Supplement: Supplemental Information 3 — (A) Gram Negative. (B) Gram Positive. (C) Potentially Pathogenic. (D) Stress Tolerant. SZ, SM, and SC represent Salix zangica, Salix myrtilllacea, and Salix cheilophila samples, respectively. [file peerj-13-19182-s003.pdf]
