# Supplementary material for: A comparative analysis of the rhizosphere microbial communities among three species of the Salix genus
Source: PeerJ. 2025 Mar 28;13:e19182. doi: 10.7717/peerj.19182 (PMC11956769; doi:10.7717/peerj.19182)
Supplement: Supplemental Information 4 [file peerj-13-19182-s004.docx]

Table S1 The symbiotic modes from different *Salix* species samples

| Guild | Trophic Mode | SM | SC | SZ |
| --- | --- | --- | --- | --- |
| Arbuscular Mycorrhizal | Symbiotroph | 20 | 3.666666667 | 1.333333333 |
| Ectomycorrhizal | Symbiotroph | 10264 | 3255.333333 | 244 |
| Endophyte | Symbiotroph | 9.666666667 | 1.666666667 | 53 |
| Epiphyte | Symbiotroph | 1.666666667 | 1 | 2 |
| Ericoid Mycorrhizal | Symbiotroph | 0.666666667 | 0 | 0 |
| Lichenized | Symbiotroph | 0 | 3.333333333 | 0 |
